# Development of capsular polysaccharide-based glycoconjugates for immunization against melioidosis and glanders

**DOI:** 10.3389/fcimb.2012.00108

**Published:** 2012-08-15

**Authors:** Mary N. Burtnick, Christian Heiss, Rosemary A. Roberts, Herbert P. Schweizer, Parastoo Azadi, Paul J. Brett

**Affiliations:** ^1^Department of Microbiology and Immunology, University of South AlabamaMobile, AL, USA; ^2^Complex Carbohydrate Research Center, The University of GeorgiaAthens, GA, USA; ^3^Department of Microbiology, Immunology and Pathology, Colorado State UniversityFort Collins, CO, USA

**Keywords:** *Burkholderia pseudomallei*, *Burkholderia mallei*, capsular polysaccharide, glycoconjugate, vaccine, immunization

## Abstract

*Burkholderia pseudomallei* and *Burkholderia mallei*, the etiologic agents of melioidosis and glanders, respectively, cause severe disease in humans and animals and are considered potential agents of biological warfare and terrorism. Diagnosis and treatment of infections caused by these pathogens can be challenging and, in the absence of chemotherapeutic intervention, acute disease is frequently fatal. At present, there are no human or veterinary vaccines available for immunization against these emerging/re-emerging infectious diseases. One of the long term objectives of our research, therefore, is to identify and characterize protective antigens expressed by *B. pseudomallei* and *B. mallei* and use them to develop efficacious vaccine candidates. Previous studies have demonstrated that the 6-deoxy-heptan capsular polysaccharide (CPS) expressed by these bacterial pathogens is both a virulence determinant and a protective antigen. Consequently, this carbohydrate moiety has become an important component of the various subunit vaccines that we are currently developing in our laboratory. In the present study, we describe a reliable method for isolating CPS antigens from O-polysaccharide (OPS) deficient strains of *B. pseudomallei*; including a derivative of the select agent excluded strain Bp82. Utilizing these purified CPS samples, we also describe a simple procedure for covalently linking these T-cell independent antigens to carrier proteins. In addition, we demonstrate that high titer IgG responses can be raised against the CPS component of such constructs. Collectively, these approaches provide a tangible starting point for the development of novel CPS-based glycoconjugates for immunization against melioidosis and glanders.

## Introduction

*Burkholderia pseudomallei* and *Burkholderia mallei*, the causative agents of melioidosis and glanders, respectively, are both CDC category B select agents (Howe and Miller, 1947; Redfearn et al., 1966; Yabuuchi et al., 1992). These facultative intracellular, Gram-negative pathogens are highly infectious via the respiratory route, and can cause severe, debilitating and often fatal diseases in humans and animals (Wiersinga et al., 2006; Whitlock et al., 2007; Galyov et al., 2010; Limmathurotsakul et al., 2012). The clinical symptoms of melioidosis and glanders tend to be multi-faceted and disease may manifest as chronic or acute localized infections, acute pulmonary infections, or fulminating septicemias. Treatment of these diseases can be challenging and even with appropriate chemotherapeutic intervention, mortality rates are unacceptably high (White, 2003; Khan et al., 2012; Meumann et al., 2012). At present, there are no human or veterinary vaccines available for immunization against melioidosis or glanders (Bondi and Goldberg, 2008; Sarkar-Tyson and Titball, 2010; Peacock et al., 2012).

Due to the potential misuse of *B. pseudomallei* and *B. mallei* as agents of biological warfare and terrorism, as well as their impact on public health in endemic regions, there has been a resurgence of interest in developing effective melioidosis and glanders vaccines (Rotz et al., 2002; Voskuhl et al., 2003; Peacock et al., 2012). To date, however, attempts to identify suitable candidates have been met with limited success. Recent studies indicate that vaccination of mice with lipopolysaccharide (LPS), capsular polysaccharide (CPS), non-viable (heat-killed/irradiated), or live-attenuated preparations of *B. pseudomallei* or *B. mallei* provide varying degrees of protection against a lethal challenge (Atkins et al., 2002; Nelson et al., 2004; Stevens et al., 2004; Ulrich et al., 2005; Whitlock et al., 2008; Sarkar-Tyson et al., 2009; Norris et al., 2011). Interestingly, whole cell preparations that fail to protect mice against challenge all appear to stimulate either mixed T-helper 1 (Th1)/T-helper 2 (Th2)-like or Th2-like biased cellular and humoral responses (Amemiya et al., 2002; Ulrich et al., 2005; Amemiya et al., 2006). In contrast, preparations affording protection appear to stimulate Th1-like biased cytokine (IL-2 and IFN-γ) and immunoglobulin responses (IgG2a>IgG1) (Ulrich et al., 2005; Amemiya et al., 2006). Based upon these findings, immunity to melioidosis and glanders appears to be complex, requiring both humoral and cell-mediated responses. In addition, these studies suggest that whole cell or subunit based vaccine candidates promoting Th1-like responses will likely be required to immunize against disease caused by *B. pseudomallei* and *B. mallei*.

Capsular polysaccharides are a major component of Gram-negative cell envelopes. Structurally diverse in nature, these highly hydrated antigens exhibit a variety of important biological functions including resistance to desiccation, adherence to host tissues and protection against innate/acquired immune defenses (Bazaka et al., 2011). Curiously, *B. pseudomallei* and *B. mallei* isolates appear to be capable of expressing only a limited repertoire of structurally diverse CPS antigens. In fact, it has even been suggested that virulent clinical and/or environmental isolates of these pathogens can be defined by a single CPS serotype (Zou et al., 2008; Sim et al., 2010). At present, the significance of these observations with regard to virulence and evasion of host immune responses remains to be fully determined. Nevertheless, this attribute certainly bodes well from a vaccine development standpoint.

Previous studies have demonstrated that the predominant CPS antigen expressed by *B. pseudomallei* is an unbranched homopolymer consisting of monosaccharide repeats having the structure -3)-2-*O*-acetyl-6-deoxy-β-D-*manno*-heptopyranose-(1- (6-deoxy-heptan; Perry et al., 1995). Although it has been recently shown that *B. mallei* also expresses an identical CPS antigen, studies indicate that for the most part, isolates of the closely related but non-pathogenic species, *Burkholderia thailandensis*, do not (Brett et al., 1998; DeShazer et al., 2001; Reckseidler et al., 2001). Based upon these observations, it has been proposed that the relative avirulence of *B. thailandensis*, in comparison to *B. pseudomallei* and *B. mallei*, may be due in part to the absence of this carbohydrate antigen (DeShazer et al., 2001; Reckseidler et al., 2001; Galyov et al., 2010).

Virulent isolates of *B. pseudomallei* and *B. mallei*, whether of human or veterinary origin, all appear to express the 6-deoxy-heptan CPS antigen (Zou et al., 2008). Importantly, previous studies by Reckseidler et al. (2001) have demonstrated that CPS-deficient mutants of *B. pseudomallei* are avirulent in a hamster model of infection. Similarly, studies by DeShazer et al. (2001) have also demonstrated that CPS-deficient mutants of *B. mallei* are avirulent in both murine and hamster models of infection. More recently, it has been shown that capsule production by *B. pseudomallei* contributes to resistance to phagocytosis by reducing C3b deposition on the surface of the bacterium, again implicating CPS as important virulence determinant (Reckseidler-Zenteno et al., 2005). Additionally, and germane to the present study, it has been shown that murine monoclonal antibodies (mAbs) specific for CPS are capable of passively immunizing mice against lethal challenges of *B. pseudomallei* and *B. mallei* (Jones et al., 2002; Zhang et al., 2011; AuCoin et al., 2012). Such findings confirm the protective capacity of this surface exposed antigen and support the rationale for exploring the use of CPS-based glycoconjugates as melioidosis and glanders vaccine candidates.

In this study, we describe the use of genetic, chemical, physical, and immunological approaches to facilitate the development and preliminary testing of *B. pseudomallei* CPS-based glycoconjugates. It is anticipated that via the application of these approaches, we will gain valuable insights toward the rational design of glycoconjugate vaccines for immunization against diseases caused by *B. pseudomallei* and *B. mallei*.

## Materials and methods

### Strains and growth conditions

The bacterial strains used in this study are described in Table 1. Unless otherwise stated, *B. pseudomallei*, *B. mallei*, and *E. coli* were grown at 37°C on Luria Bertani-Lennox (LBL) agar or in LBL broth. For *B. pseudomallei* Bp82 and its derivatives, growth media were supplemented with 100 μg/ml adenine hydrochloride (Sigma) and 5 μg/ml thiamine hydrochloride (Sigma). When appropriate, antibiotics were added at the following concentrations: 25 μg/ml kanamycin (Km) or 15 μg/ml polymyxin B (Pm) for *E. coli* and 100 μg/ml (DD503) or 500 μg/ml (Bp82) Km for *B. pseudomallei*. Bacterial stocks were maintained at –80°C as 20% glycerol suspensions. Other than *B. pseudomallei* Bp82 and its derivatives, all studies with *B. pseudomallei* and *B. mallei* were conducted in a CDC select agent certified biosafety level 3 containment facility.

**Table 1:** **Strains, plasmids and primers**.

**Strains**	**Relevant characteristics**	**Source or reference**
***E. coli***		
TOP10	General cloning strain: Km^s^, Pm^s^	Invitrogen
S17-1	Mobilizing strain: Km^s^, Pm^s^	Simon et al., 1983
***B. pseudomallei*^a^**		
1026b	Clinical isolate	DeShazer et al., 1997
DD503	1026b derivative; Δ(*amrR-amrAB-oprA*): Km^s^, Pm^r^	Moore et al., 1999
BP2683	DD503 derivative; Δ*rmlD*: Km^s^, Pm^r^	Heiss et al., 2012
Bp82	1026b derivative; Δ*purM*: Km^r^, Pm^r^	Propst et al., 2010
RR2683	Bp82 derivative; Δ*rmlD*: Km^r^, Pm^r^	This study
***B. mallei***		
ATCC 23344	Type strain; isolated in 1944 from a human case of glanders	Yabuuchi et al., 1992
**PLASMIDS**		
pMo130	Gene replacement vector; *oriT sacB*: Km^r^	Hamad et al., 2009
pMoΔ*rmlD*	pMo130 harboring *rmlD* with an internal 354-bp deletion: Km^r^	This study
**PCR PRIMERS^b^**		
BprmlD-5FH	5′-CATG*AAGCTT*TTCAGGACAATCACTCCCGATCCG-3′	This study
BprmlD-5RPs	5′-TTGATCACATTCG*CTGCAG*CACCATC-3′	This study
BprmlD-3FPs	5′-GCGATCGATTG*CTGCAG*GCACGGCCCA-3′	This study
BprmlD-3RXb	5′-CATG*TCTAGA*GACACATTCGGCGAACAATCCATG-3′	This study

a The majority of prototype B. pseudomallei strains are resistant to high levels of Km due to expression of the AmrAB-OprA efflux pump. For genetic manipulations this intrinsic resistance can be overcome using high (>500 μg/ml) Km concentrations.

b Restriction sites are italicized.

### Recombinant DNA techniques

The plasmids and oligonucleotide primers used in this study are described in Table 1. DNA manipulations were performed using standard methods. Restriction enzymes and T4 DNA Ligase (New England BioLabs) were used according to manufacturer's instructions. PCR was performed using an Expand High Fidelity PCR System (Roche Applied Science) or GoTaq DNA Polymerase (Promega); 1 M Betaine (Sigma) was included in all PCR reactions. PCR was performed using the following conditions: 97°C for 5 min; 30 cycles, each consisting of 97°C for 45 s, 55°C for 45 s, and 72°C for 3 min; a final extension step of 72°C for 10 min was included. PCR and restriction digested products were purified using a QIAquick Gel Extraction Kit (Qiagen). Plasmids were purified using a QIAprep Spin Miniprep Kit (Qiagen). Genomic DNA was purified using a Wizard Genomic DNA Purification kit (Promega). Chemically competent *E. coli* TOP10 cells were transformed as per the manufacturer's instructions (Invitrogen). Oligonucleotide primers were obtained from Integrated DNA Technologies (Coralville, IA).

### Mutant construction

Gene replacement experiments with *B. pseudomallei* were conducted using the *sacB*-based vector pMo130. To construct an in-frame deletion in the O-polysaccharide (OPS)-related biosynthetic gene, *rmlD* (BPSL2683), the rmlD-5FH/rmlD-5RPs and rmlD-3FPs/rmlD-3RXb primer pairs were used to PCR amplify ~600 bp DNA fragments upstream (rmlD5′) and downstream (rmlD3′) of the gene, respectively. The rmlD5′ PCR product was digested with HindIII and PstI and cloned into pMo130 digested with the same enzymes resulting in plasmid pMormlD5′. The rmlD3′ PCR product was then digested with PstI and XbaI and cloned into pMormlD5′ digested with the same enzymes. The resulting plasmid was designated pMoΔrmlD (harboring *rmlD* with a 354-bp deletion, Δ*rmlD*).

To construct the *B. pseudomallei* OPS mutant strains BP2683 and RR2683, *E. coli* S17-1 was used to mobilize pMoΔrmlD into *B. pseudomallei* DD503 or Bp82 via conjugative mating essentially as previously described (DeShazer et al., 1997; Burtnick et al., 2011). Briefly, overnight cultures of S17-1 (pMoΔrmlD) and DD503 or Bp82 were pelleted by centrifugation, resuspended together in 100 μl of 10 mM MgSO_4_, spotted onto LB agar plates and incubated for 18 h at 37°C. To select for transconjugants, mating mixtures were plated onto LB-Km-Pm agar and incubated at 37°C for 48 h. To select for sucrose resistant colonies, individual transconjugants were inoculated into yeast-tryptone (YT) broth, incubated at 37°C for 4–5 h, and then plated onto YT agar containing 15% sucrose (Hamad et al., 2009). Following incubation at 37°C for 48 h, sucrose resistant colonies were screened for loss of the Km resistance marker by replica plating onto LB and LB-Km. The resolved co-integrates were screened for the presence of the mutant allele (Δ*rmlD*) by PCR.

### CPS purification

LBL broth (4 × 600 ml in 2 L baffled Erlenmeyer flasks) was inoculated with *B. pseudomallei* BP2683 or RR2683 and incubated overnight at 37°C with vigorous shaking. Cell pellets were obtained by centrifugation (10 min at 8000 × *g*) and extracted using a modified hot aqueous-phenol procedure (Perry et al., 1995). Following extraction, the resulting phenol and aqueous phases were combined and dialyzed against distilled water to remove the phenol. The dialysates were then clarified by centrifugation (20 min at 10,000 × *g*) and the supernatants concentrated by lyophilization. The crude CPS preparations were solubilized at 20 mg/ml in RD buffer [10 mM Tris-HCl (pH 7.5), 1 mM MgCl_2_, 1 mM CaCl_2_, 50 μg/ml RNase A and 50 μg/ml DNase I] and incubated for 3 h with gentle shaking at 37°C. Proteinase K was then added to a final concentration of 50 μg/ml and the samples were incubated for an additional 3 h at 60°C after which the enzymatic digests were clarified by centrifugation (20 min at 10,000 × *g*). CPS was then isolated from the supernatants as precipitated gels following successive rounds of ultracentrifugation (3 × 2 h at 100,000 × *g* with the pellets being resuspended in ultrapure water between spins). After the final spin, the gel-like pellets were resuspended in ultrapure water and lyophilized.

To remove the rough LPS contaminants from the CPS preparations, crude samples were solubilized at 5 mg/ml in 2% acetic acid and incubated for 2 h at 100°C. The hydrolyzed samples were cooled to room temperature and clarified via centrifugation (20 min at 10,000 × *g*) following which the supernatants were carefully removed and lyophilized to concentrate. The lyophilized samples were then solubilized at 20 mg/ml in 100 mM phosphate buffered saline (pH 7.4; PBS) and clarified using 0.45 μm syringe filters. The samples were loaded onto Sephadex G-50 columns (40 × 2.6 cm) equilibrated with PBS and eluted with the same buffer. Fractions (5 ml) were collected and assayed for carbohydrate using the phenol-sulfuric acid method (Dubois et al., 1956). Carbohydrate positive fractions eluting near the column void volumes (~70 ml) were pooled, extensively dialyzed against distilled water and lyophilized. Protein and nucleic acid contamination of the resulting CPS preparations were estimated by BCA assay (Pierce) and A_260/280_ measurements, respectively.

### NMR spectrometry

Column purified CPS was deuterium exchanged by dissolving in D_2_O followed by lyophilization. Samples were then dissolved in D_2_O containing a trace amount of acetone and ^1^H and ^13^C NMR spectra were obtained using a Varian Inova-500 MHz spectrometer at 50°C using standard pulse sequences. ^1^H and ^13^C chemical shifts were measured relative to the internal acetone reference (δ_H_ = 2.218 ppm; δ_C_ = 33.0 ppm) (Wishart et al., 1995).

### Glycoconjugate synthesis

Glycoconjugates were synthesized using established methodologies (Brett and Woods, 1996; Jennings and Lugowski, 1981; Conlan et al., 2002). Briefly, purified CPS was solubilized at 5 mg/ml in PBS and added to a small amber vial. To each ml of the solution was added 6 mg (~30 mM) of sodium *meta*-periodate (NaIO_4_; Pierce). Once the crystals had dissolved by gentle agitation, the reaction mixture was gently stirred at room temperature for 40 min. To remove any excess oxidizing agent, the reaction mixture was applied to a Zeba Desalt Spin Column (Pierce) equilibrated with PBS and the eluate collected. To facilitate conjugation of the CPS to cationized bovine serum albumin (cBSA; Pierce), the activated CPS was added to small amber vials. To each ml of the CPS solutions was added either 0.5, 1, or 2 ml of the carrier protein (5 mg/ml in PBS). Following mixing by gentle agitation, 10 μl aliquots of a sodium cyanoborohydride stock (1 M NaBH_3_CN in 10 mM NaOH) were added to each ml of the conjugation mixtures and the reactions were gently stirred at room temperature for 4 d. Following this, 10 μl aliquots of a sodium borohydride stock (1 M NaBH_4_ in 10 mM NaOH) were added to each ml of the conjugation mixtures and the reactions were stirred for 40 min. The conjugate reactions were then brought to 5 ml with ultrapure water, dialyzed against distilled water and then lyophilized. The resulting preparations were resuspended in ultrapure water as 1 mg/ml stocks and stored at −20°C until required for use. BCA assays were used to quantitate the protein concentrations of the glycoconjugate stocks.

### Antibody production

Standard immunization protocols were used at Cocalico Biologicals, Inc. to generate mouse immune serum samples. Briefly, 6–8 week old female BALB/c mice were immunized subcutaneously on days 0, 14, 21, and 49 with 10 μg of the CPS2B1 glycoconjugate formulated using TiterMax as an adjuvant. Unconjugated CPS and cBSA antigens served as controls. Terminal bleeds were conducted 7 days after the last immunization. All procedures involving the mice were performed according to protocols approved by the Cocalico Biologicals, Inc. Animal Care and Use Committee.

### SDS-PAGE and western immunoblotting

Glycoconjugate samples were solubilized in 1X SDS-PAGE sample buffer and heated to 100°C for 5 min prior to electrophoresis on 12% Precise gels (Pierce). Proteins were visualized via staining with Coomassie Blue R-250. Whole cell lysates were prepared from *B. pseudomallei* 1026b and *B. mallei* ATCC 23344 essentially as previously described (Brett et al., 2011). For immunoblot analyses, treated whole cell lysates or the glycoconjugate samples were separated on 12% Precise gels and electrophoretically transferred to nitrocellulose membranes. The membranes were blocked with 3% skim milk in high salt Tris-buffered saline (HS-TBS; 20 mM Tris, 500 mM NaCl, pH 7.5) for 60 min at room temperature and then incubated overnight at 4°C with 1/1000 dilutions of pooled anti-CPS2B1 immune serum or the CPS-specific mAb, 3C5 (Nuti et al., 2011). To facilitate detection, the membranes were incubated for 1 h at room temperature with 1/5000 dilutions of an anti-mouse IgG horse radish peroxidase conjugate (SouthernBiotech). The blots were then visualized using HRP Color Development Reagent (Bio-Rad) or Pierce ECL Western Blotting Substrate (Pierce).

### Quantitation of immunoglobulin titers by ELISA

Ninenty-six-well Maxisorp plates (Nunc) were coated overnight at 4°C with purified CPS (10 μg/ml) solubilized in carbonate buffer (pH 9.6). The plates were blocked at room temperature for 30 min with StartingBlock T20 (TBS) Blocking Buffer (Pierce) and then incubated for 2 h at 37°C with the mouse serum samples serially diluted in Tris-buffered saline + 0.05% Tween 20 (TBS-T; pH 7.5) + 10% StartingBlock T20. To facilitate detection, the plates were incubated for 1 h at 37°C with 1/2000 dilutions of an anti-mouse IgG horse radish peroxidase conjugate (SouthernBiotech). The plates were then developed with TMB substrate (KPL) and read at 620 nm. The reciprocals of the highest dilutions exhibiting ODs of >0.150 were used to determine the endpoint titers for the individual mice. The data was plotted and analyzed using GraphPad Prism 5 (GraphPad Software Inc.). Statistical differences between geometric mean IgG titers was assessed by Mann–Whitney rank sum analysis with the significance set at *P* < 0.05.

### Cell culture and opsonophagocytosis assays

The murine macrophage cell line RAW 264.7 (ATCC TIB-71) was maintained in Dulbecco's modified Eagle's medium supplemented with 10% (v/v) heat inactivated (HI) fetal bovine serum (DMEM-10; Invitrogen) and a standard mixture of antibiotics (100 U ml^−1^ penicillin, 100 μ g ml^−1^ streptomycin and 250 μg ml^−1^ amphotericin B; Sigma) at 37°C under an atmosphere of 5% CO_2_. Opsonophagocytosis assays were based upon previously described methods (Simon et al., 2011; Tennant et al., 2011). Briefly, RAW 264.7 cells resuspended in DMEM-10 were transferred into 24-well tissue culture plates at a density of ~1 × 10^6^ cells/well and incubated overnight. *B. mallei* cultures grown to early-log phase were pelleted, resuspended at a density of ~10^6^ cfu/ml in DMEM or DMEM containing 0.2% cBSA or CPS2B1 mouse immune serum (pooled and HI for 30 min at 56°C) and then incubated at 37°C for 1 h. RAW 264.7 monolayers were washed twice with Hanks' Balanced Salts Solution (HBSS; Invitrogen) prior to the addition of the opsonized bacterial suspensions. The monolayers were incubated with the bacteria at 37°C under an atmosphere of 5% CO_2_ for 1 h and then washed twice with HBSS to remove extracellular bacteria. Infected RAW 264.7 cells were incubated in fresh DMEM-10 containing 250 μg/ml km to suppress the growth of residual extracellular bacteria. At 3 h post-infection, monolayers were washed twice with HBSS, lysed with 0.2% (v/v) Triton X-100 (Sigma) and serial dilutions of the lysates were plated onto LB agar supplemented with 4% glycerol and incubated at 37°C for 48 h. Plate counts were used to enumerate bacterial loads. The data was plotted and analyzed using GraphPad Prism 5. Statistical differences were assessed by *t*-test with the significance set at *P* < 0.05.

## Results and discussion

### Purification and characterization of *B. pseudomallei* CPS antigens

**Figure 1:**
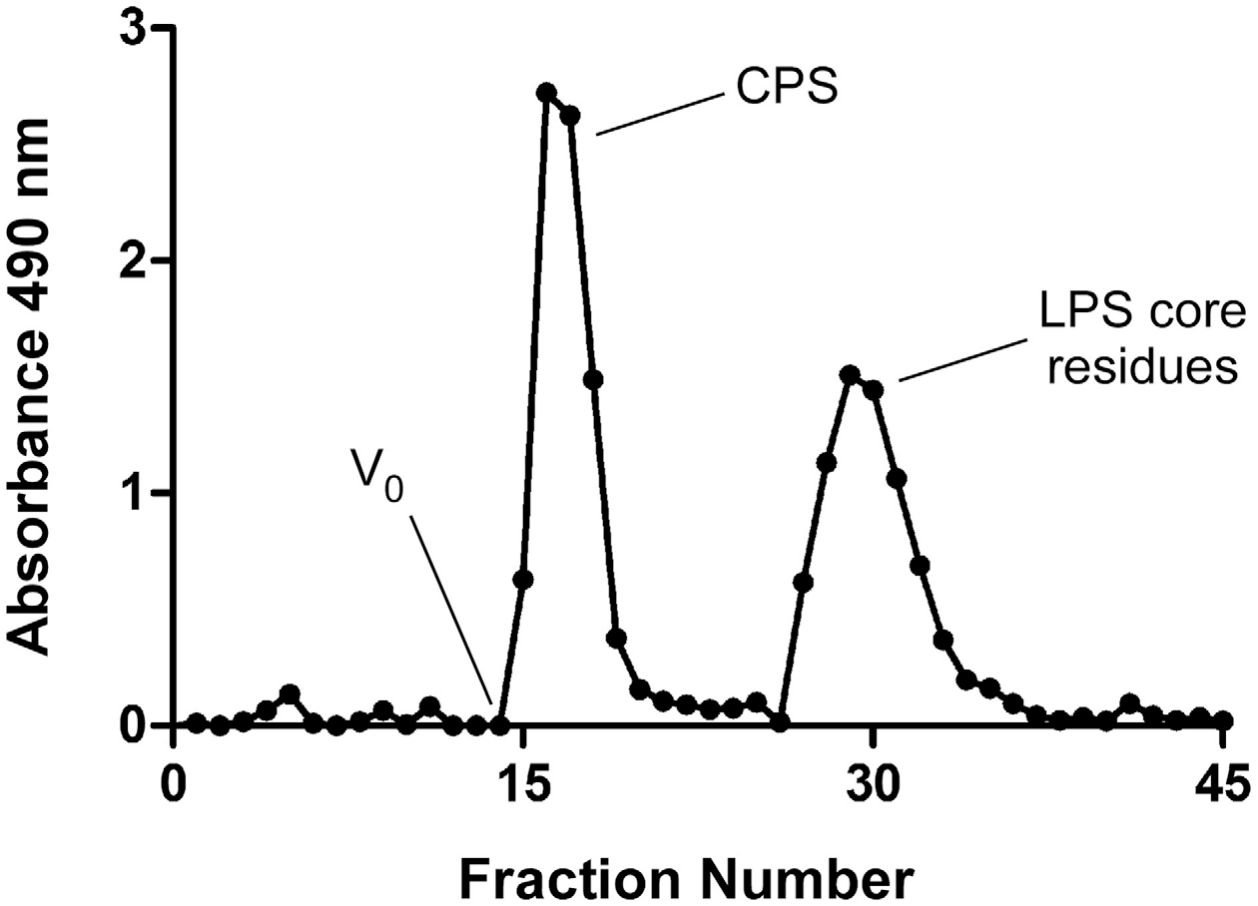
**Elution profile of column purified *B. pseudomallei* CPS**. Acid hydrolyzed BP2683 CPS was loaded onto a Sephadex G-50 column and eluted with PBS. Carbohydrate positive fractions eluting near the column void volumes (V_o_) were collected and pooled for further use.

Previous studies by Perry et al. (1995) have shown that a modified enzyme hot aqueous-phenol procedure can be used to isolate OPS and CPS antigens from wild type strains of *B. pseudomallei*. Since both of these high molecular weight species partition into the phenol phase during the extraction step, separation of these antigens from one another during downstream purification can be challenging. Fortunately, studies have demonstrated that highly purified preparations of CPS can be obtained from naturally occurring isolates of *B. pseudomallei* expressing rough LPS antigens (Perry et al., 1995). Based upon these observations, we used allelic exchange mutagenesis to construct the OPS-deficient mutant, *B. pseudomallei* BP2683, to help facilitate the development of our CPS-based glycoconjugates (Heiss et al., 2012). Utilizing this strain, we found that we could obtain highly purified preparations of CPS from *B. pseudomallei* since lower molecular weight contaminants (e.g., rough LPS) rather than higher molecular weight contaminants (e.g., smooth LPS) could be easily separated from the homopolymer by size exclusion chromatography (Figure 1). Recently, we have also been successful in isolating CPS from *B. pseudomallei* RR2683, an OPS-deficient derivative of the select agent excluded strain Bp82 (Propst et al., 2010). By using this mutant strain, we now have the ability to more safely and cost-effectively purify CPS from *B. pseudomallei* without the requirement for BSL-3 containment which should help to accelerate the pace of our research.

**Figure 2:**
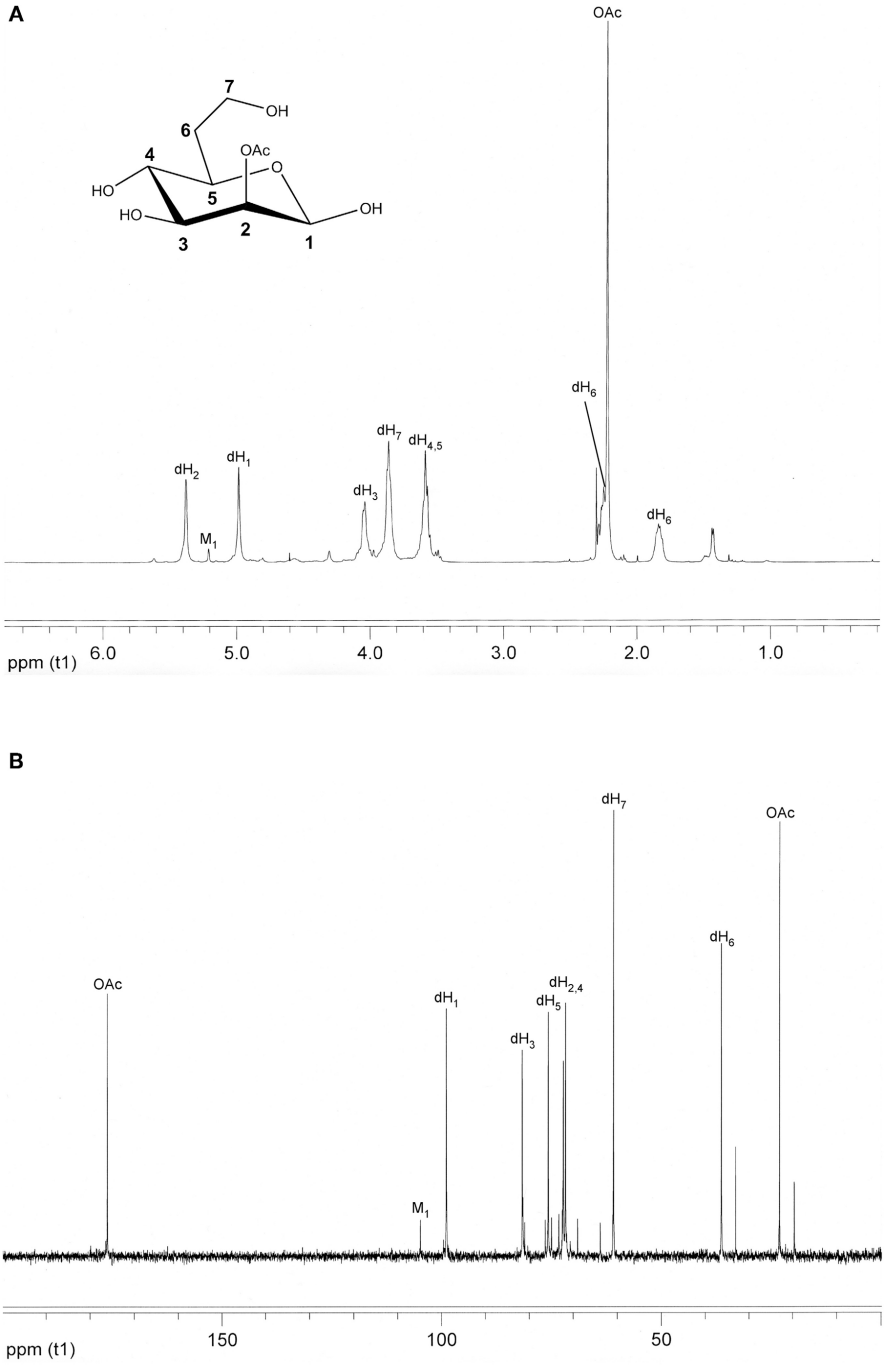
**Structural analysis of *B. pseudomallei* CPS**. **(A)** ^1^H NMR and **(B)** ^13^C NMR spectra were obtained for column purified BP2683 CPS using a Varian Inova-500 MHz spectrometer. For reference purposes, the chemical structure of the monosaccharide making up the CPS homopolymer is inset in panel **A**. dH, 6-deoxy-heptose; M, mannose; OAc, *O*-acetyl.

Using the purification strategy outlined in the “Materials and Methods” section, we routinely obtain 10–15 mg of CPS per liter of BP2683 or RR2683 culture with the resulting antigen preparations being devoid of any detectable protein or nucleic acid contamination as determined by BCA assay or UV spectroscopy. To assess the structural integrity and homogeneity of the CPS preparations, samples are also analyzed by NMR spectrometry. Using this approach, ^1^H and ^13^C NMR analyses confirm that the main component of these preparations is the desired 2-*O*-acetylated 6-deoxy-heptan homopolymer (Figures 2A,B). In addition, such analyses indicate that the preparations are ~95% pure with the remainder of the samples consisting of the unique mannan CPS recently discovered by Heiss et al. (2012).

### Conjugation of CPS to carrier proteins

**Figure 3:**
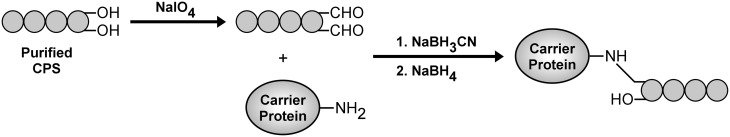
**Generalized scheme for the conjugation of activated *B. pseudomallei* CPS to carrier proteins**.

To facilitate the coupling of *B. pseudomallei* CPS to protein carriers, we have found that NaIO_4_ is well suited for chemically activating the polysaccharide. The decision to use NaIO_4_ was based upon several considerations including (1) the absence of amines or carboxylates in the antigen that would favor conjugation via other methods, (2) the relative safety of the compound over alternatives such as cyanogen bromide, (3) the use of reaction conditions to minimize the risk of reducing alkali-sensitive *O*-acetyl modifications, (4) the desire to construct neoglycoconjugates rather than cross-linked network conjugates (Jones, 2005). Using the approach outlined in Figure 3, reactive aldehydes incorporated into the reducing/non-reducing termini of *B. pseudomallei* CPS facilitate the conjugation of the antigen to carrier proteins via reductive amination. To reduce the Schiff bases formed during the coupling reactions, NaBH_3_CN was chosen as the primary reducing reagent because, unlike NaBH_4,_ it rapidly modifies Schiff bases but not aldehydes. Once the CPS moieties are coupled to carrier proteins, NaBH_4_ is then added to quench any residual aldehydes in the reaction mixtures.

**Figure 4:**
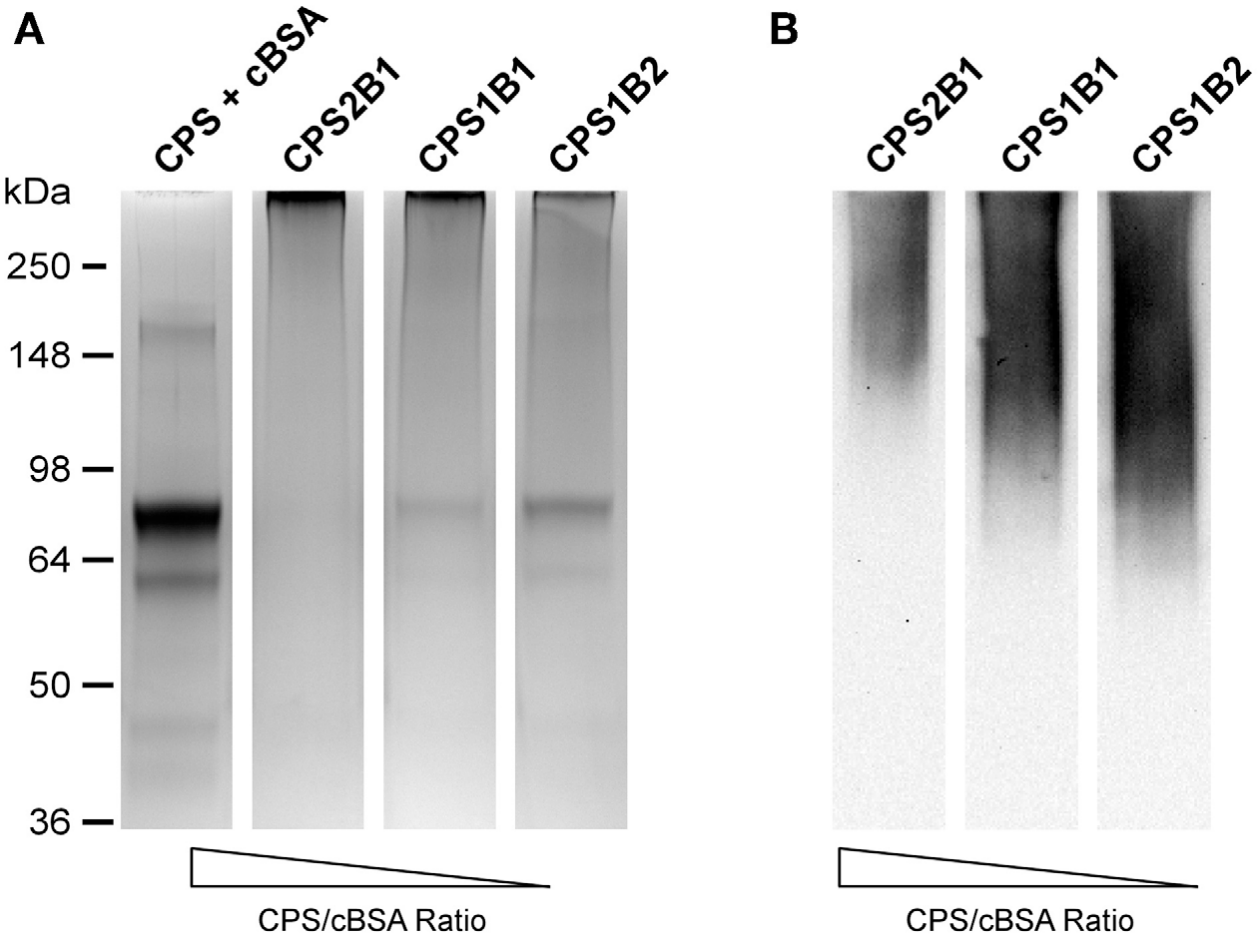
**Physical analysis of *B. pseudomallei* CPS-cBSA glycoconjugates**. **(A)** SDS-PAGE and Coomassie Blue staining was used to confirm the covalent linkage of BP2683 CPS to cBSA. The positions of protein molecular size standards are indicated on the left. **(B)** Western immunobloting was also used to assess the antigenicity of the chemically activated/coupled BP2683 CPS. The CPS + cBSA lane represents unconjugated controls. CPS was detected using the 3C5 mAb. Lanes were loaded with similar amounts of protein or carbohydrate to facilitate direct comparisons.

The CPS-based glycoconjugates synthesized in this study were constructed using cBSA as the protein carrier. The decision to use cBSA for this purpose was based on several considerations, including the fact that it is (1) a well characterized immunogen, (2) commercially available, and (3) affordable. To facilitate our studies, glycoconjugates were synthesized by reacting various ratios of chemically activated CPS and cBSA with one another (e.g., 2:1, 1:1, and 1:2 ratios of CPS:cBSA). Following conjugation of the antigens, the samples were examined by SDS-PAGE. Results of these analyses indicated that in all instances, the CPS had covalently linked to the protein carrier as demonstrated by the shift in molecular weight of the cBSA relative to the unconjugated control (Figure 4A). In addition, Western immunobloting confirmed that the structural integrity/antigenicity of the CPS moieties remained intact following chemical activation and linkage to the protein carrier based upon their reactivity with the 3C5 mAb (Figure 4B). Furthermore, studies demonstrated that by varying initial carbohydrate to protein ratios, we could influence the molecular weights of the resulting glycoconjugates.

### Immune responses against CPS-based glycoconjugates

Efforts to overcome the poor immunogenicity of many polysaccharides has led to the development of conjugate vaccines (Lockhart, 2003). Covalent linkage of polysaccharides to carrier proteins enables them to stimulate humoral immune responses characteristic of T cell-dependent antigens. By recruiting Th cell involvement, immunological memory is evoked, isotype switching occurs and complement-activating/opsonizing antibody isotypes are generated. The avidities of the antibodies elicited by glycoconjugates are also much higher than those produced by polysaccharides alone (Kuberan and Linhardt, 2000; Lesinski and Westerink, 2001; Weintraub, 2003).

**Figure 5:**
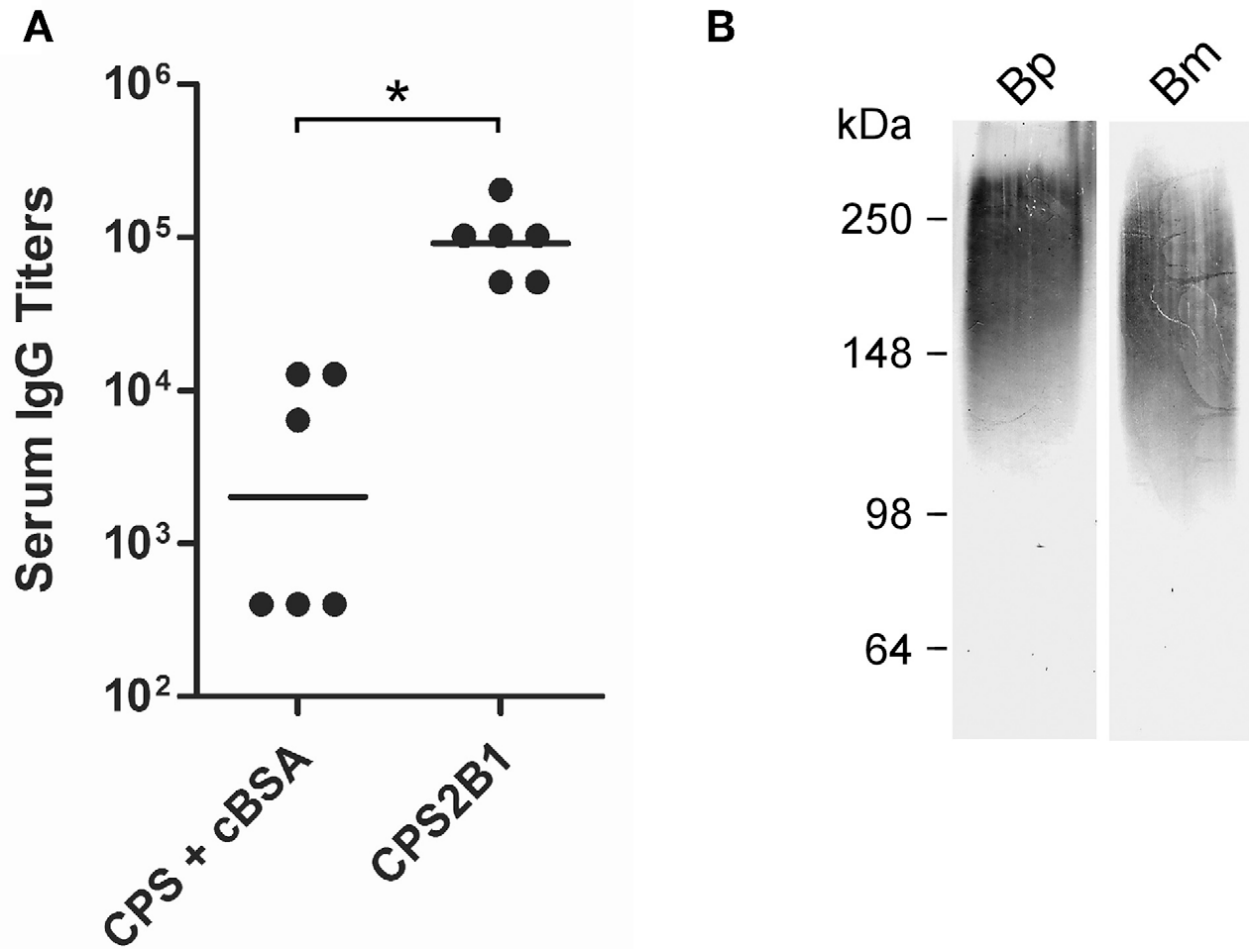
**Characterization of murine immune responses against the CPS2B1 conjugate**. **(A)** ELISA was used to determine the total serum IgG response of mice immunized with CPS2B1 or the unconjugated controls. Individual symbols represent a single mouse while black bars indicate geometric means. ^*^, *P* < 0.05. **(B)** Western immunoblotting was used to assess the reactivity of pooled anti-CPS2B1 immune serum (*n* = 6) with *B. pseudomallei* 1026b (Bp) and *B. mallei* ATCC 23344 (Bm) whole cell lysates. The positions of protein molecular size standards are indicated on the left.

**Figure 6:**
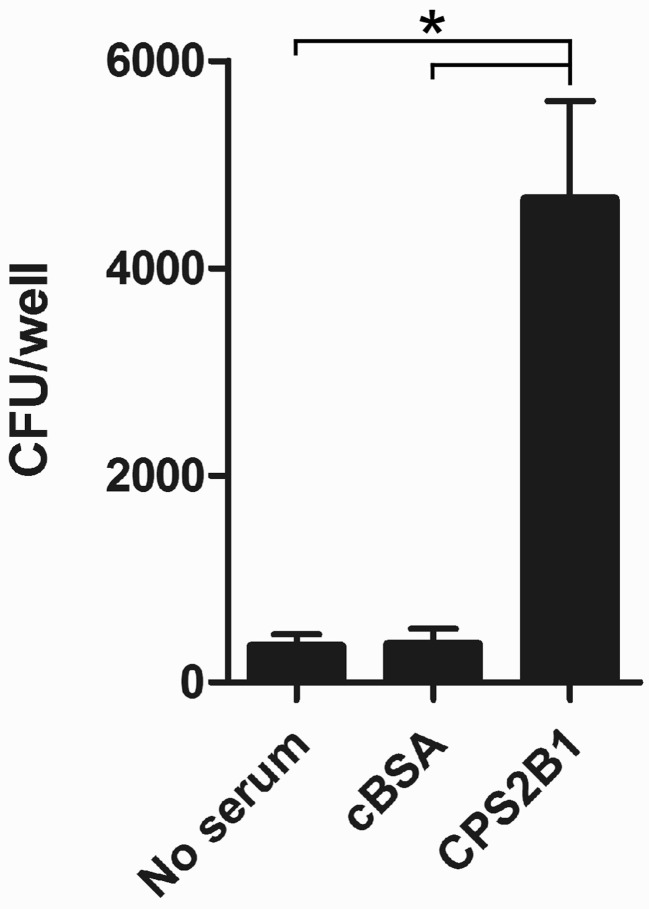
**Opsonophagocytic uptake of *B. mallei* by RAW 264.7 murine macrophages**. Bacterial uptake in the presence of DMEM alone (no serum), pooled HI anti-cBSA immune serum (cBSA; *n* = 6) or pooled HI anti-CPS2B1 immune serum (CPS2B1; *n* = 6) was quantitated at 3 h post-infection. Values represent the means ± SD of three independent experiments. ^*^, *P* < 0.05.

To examine the immunogenic potential of the glycoconjugates synthesized in this study, mice were immunized with the CPS2B1 construct (37% protein on a w/w basis) as well as unconjugated controls. Analysis of the resulting serum samples indicated that mice immunized with the glycoconjugate exhibited significantly higher anti-CPS IgG titers (45-fold) than those immunized with the unconjugated control (Figure 5A). To further examine the immune response raised against CPS2B1, mouse serum samples were pooled and reacted against proteinase K treated whole cell lysates of *B. pseudomallei* and *B. mallei* by Western immunoblotting. As anticipated, results demonstrated that the antiserum reacted strongly with CPS antigens expressed by both pathogens (Figure 5B). In addition, the CPS2B1 antiserum was examined for its ability to mediate opsonophagocytic uptake. As shown in Figure 6, pre-incubation of *B. mallei* with pooled anti-CPS2B1 serum, but not pooled anti-cBSA serum, significantly enhanced bacterial uptake into RAW 264.7 murine macrophages.

## Conclusions

Melioidosis and glanders are emerging/re-emerging infectious diseases for which no licensed vaccines currently exist. Several recent reports have demonstrated that mAbs specific for the 6-deoxy-heptan CPS expressed by *B. pseudomallei* and *B. mallei* are passively protective in animal models of infection (Jones et al., 2002; Nelson et al., 2004; Zhang et al., 2011; AuCoin et al., 2012). Because of this, research in our lab is focused on developing CPS-based vaccines to immunize humans and animals against diseases caused by these bacterial pathogens. In the present study, we have detailed for the first time, methodologies to facilitate the isolation and structural validation of highly purified preparations of CPS from various *B. pseudomallei* 1026b derivatives. In addition, we have described a simple strategy for covalently linking these carbohydrate antigens to carrier proteins. Using these approaches, we have also demonstrated that high titer IgG responses can be raised against the carbohydrate component of the CPS-based glycoconjugates. Based upon these observations, studies are currently underway to assess the protective capacity of our prototype glycoconjugates in animal models of infection.

Because of obvious limitations regarding the use of cBSA as a carrier for vaccine development, studies are ongoing to explore the use of licensed carriers (e.g., CRM197, CtxB, ExoA etc.) or specific *Burkholderia* proteins (e.g., LolC, Hcp1, BipD etc.) for this purpose. When doing so, it will be important to evaluate if coupling of the CPS to the different carrier proteins affects their stability and immunogenicity. We anticipate that approaches such as ELISA, circular dichroism spectroscopy and protein NMR spectrometry will be required to assess these types of issues. Additionally, studies are being planned to investigate how differing adjuvants and dosing regimens might influence immune responses raised against our glycoconjugates. Collectively, it is anticipated that such studies will further provide important insights toward the rational design of efficacious melioidosis and glanders vaccine candidates.

### Conflict of interest statement

The authors declare that the research was conducted in the absence of any commercial or financial relationships that could be construed as a potential conflict of interest.
